# Detection and quantification of angiogenesis in experimental valve disease with integrin-targeted nanoparticles and 19-fluorine MRI/MRS

**DOI:** 10.1186/1532-429X-10-43

**Published:** 2008-09-25

**Authors:** Emily A Waters, Junjie Chen, John S Allen, Huiying Zhang, Gregory M Lanza, Samuel A Wickline

**Affiliations:** 1 Department of Medicine, Washington University School of Medicine, St. Louis, MO, USA; 2 Department of Biomedical Engineering, Washington University in St. Louis, St. Louis, MO, USA

## Abstract

**Background:**

Angiogenesis is a critical early feature of atherosclerotic plaque development and may also feature prominently in the pathogenesis of aortic valve stenosis. It has been shown that MRI can detect and quantify specific molecules of interest expressed in cardiovascular disease and cancer by measuring the unique fluorine signature of appropriately targeted perfluorocarbon (PFC) nanoparticles. In this study, we demonstrated specific binding of α_ν_β_3 _integrin targeted nanoparticles to neovasculature in a rabbit model of aortic valve disease. We also showed that fluorine MRI could be used to detect and quantify the development of neovasculature in the excised aortic valve leaflets.

**Methods:**

New Zealand White rabbits consumed a cholesterol diet for ~180 days and developed aortic valve thickening, inflammation, and angiogenesis mimicking early human aortic valve disease. Rabbits (n = 7) were treated with α_ν_β_3 _integrin targeted PFC nanoparticles or control untargeted PFC nanoparticles (n = 6). Competitive inhibition *in vivo *of nanoparticle binding (n = 4) was tested by pretreatment with targeted nonfluorinated nanoparticles followed 2 hours later by targeted PFC nanoparticles. 2 hours after treatment, aortic valves were excised and ^19^F MRS was performed at 11.7T. Integrated ^19^F spectral peaks were compared using a one-way ANOVA and Hsu's MCB (multiple comparisons with the best) post hoc t test. In 3 additional rabbits treated with α_ν_β_3 _integrin targeted PFC nanoparticles, ^19^F spectroscopy was performed on a 3.0T clinical scanner. The presence of angiogenesis was confirmed by immunohistochemistry.

**Results:**

Valves of rabbits treated with targeted PFC nanoparticles had 220% more fluorine signal than valves of rabbits treated with untargeted PFC nanoparticles (p < 0.001). Pretreatment of rabbits with targeted oil-based nonsignaling nanoparticles reduced the fluorine signal by 42% due to competitive inhibition, to a level not significantly different from control animals. Nanoparticles were successfully detected in all samples scanned at 3.0T. PECAM endothelial staining and α_ν_β_3 _integrin staining revealed the presence of neovasculature within the valve leaflets.

**Conclusion:**

Integrin-targeted PFC nanoparticles specifically detect early angiogenesis in sclerotic aortic valves of cholesterol fed rabbits. These techniques may be useful for assessing atherosclerotic components of preclinical aortic valve disease in patients and could assist in defining efficacy of medical therapies.

## Background

Aortic valve sclerosis is a common disease of the elderly, occurring in approximately 30% of the population aged > 65 years. Sclerosis progresses to clinically significant valvular stenosis in approximately 2% of this population [1,2]. Despite its prevalence, no medical treatment for aortic stenosis (AS) has proven effective. For severe stenosis, surgical valve replacement is the preferred treatment; however, many elderly AS patients are poor surgical candidates. Balloon valvuloplasty is a viable option for some of these patients [3], but high rates of restenosis limit its efficacy [4]. A medical treatment option would therefore greatly benefit the patient population. Recent data from a prospective study suggest that statin therapy very early in the disease course may slow its progression, but the efficacy was greatly reduced in patients who had moderate or severe valvular stenosis at the time of the study [5]. This clinical observation implies that early diagnosis of AS may be the key to developing and applying effective medical therapies.

Although AS was once considered a passive degenerative process, its pathogenesis actually involves a chronic inflammatory and immune-mediated response similar in the earliest stages to atherosclerosis. Immunohistochemistry of excised human aortic valves has demonstrated lipid deposition, endothelial damage, and infiltration by activated T-cells. Upregulation of cell adhesion molecules such as CD34, intercellular adhesion molecule 1 (ICAM-1), vascular cell adhesion molecule 1 (VCAM-1), osteopontin, and platelet and endothelial cell adhesion molecule (PECAM) has been reported [5-9]. Upregulated expression of extracellular matrix proteins such as metalloproteinases that are involved in the inflammatory response and angiogenesis also has been reported [7].

Angiogenesis appears to play a key role in this pathological process [6-9]. Angiogenesis is driven by local expression of cytokines and growth factors such as vascular endothelial growth factor (VEGF), produced by the expanding cellular infiltrate in the inflamed valve. Neovasculature serves as a conduit for the delivery of yet more inflammatory messengers and activated cells [8]. Angiogenic vasculature is distinguished from mature vasculature by marked and early upregulation of endothelial integrins (e.g. α_ν_β_3 _integrin) and adhesion molecules that are expressed in stages as the neovasculature matures.

The cholesterol-fed rabbit is a well-characterized model of atherosclerosis [10]. It has been shown that, with a slightly longer diet regimen, these rabbits develop aortic valve sclerosis [11-13]. The histological changes observed (gross thickening, macrophage infiltration, calcification, and eventual bone formation) mimic the pathobiology of human aortic stenosis [11-14]. Based on the similarities between human and rabbit disease, we hypothesized that α_ν_β_3 _integrin positive neovasculature would be present in sclerotic rabbit aortic valve leaflets, and that it could be detected noninvasively with MRI molecular imaging. Our laboratory has developed perfluorocarbon nanoparticles for MRI molecular imaging and targeted drug delivery. We have demonstrated previously that α_ν_β_3 _-integrin targeted nanoparticles bearing gadolinium chelates can be used to image angiogenesis in atherosclerotic plaques of cholesterol fed rabbits with proton MRI on a 1.5T clinical scanner [15].

In this study, we sought to demonstrate *ex vivo *proof of concept for specific nanoparticle binding to angiogenesis in the sclerotic aortic valve leaflets of cholesterol fed rabbits, and for sensitive MRI/MRS detection of fluorine in the nanoparticles. We proposed that by using the native fluorine signal emanating from the perfluorocarbon core of the particles, we could depict a unique MRI/MRS signature of angiogenesis, which is an important component of the inflammatory process [16,17]. Compared to traditional proton-based molecular imaging [18], the benefits of fluorine imaging and spectroscopy are multiple: 1) a unique and specific signature for MRI molecular imaging because ^19^F is found only in trace quantities in biological tissues, 2) lack of background signal for the same reasons, 3) no need for both pre- and post-contrast images because the signal is unique and specific, and 4) ability to perform quantitative ^19^F NMR spectroscopy and potentially estimate the concentration of molecular binding sites in a targeted sample.

## Methods

### Animal preparation

Several groups have reported that rabbits placed on an extended high-cholesterol diet develop gross thickening of the aortic valve, with histological changes including macrophage infiltration, fibrosis, and deposition of calcific nodules [11-13]. Involvement of proteins such as osteopontin and proliferating cell nuclear antigen (PCNA) characteristic of human-type disease has also been reported [14,19].

Twenty-six New Zealand White rabbits (Harlan, Indianapolis, Indiana) consumed a 0.25% cholesterol diet for 5 months to induce aortic valve disease. Normal controls consisted of four male retired breeders (Myrtle's Rabbitry, Thompson's Station, Tennessee) with similar body weights to the cholesterol-fed group (approximately 4 kg). Specimens from 17 cholesterol-fed rabbits and 3 healthy controls underwent ^19^F MRS at 11.7T, specimens from 4 cholesterol-fed rabbits underwent ^19^F MRI, specimens from 3 cholesterol-fed rabbits underwent ^19^F MRS at 3T and 11.7T, and specimens from 2 cholesterol-fed rabbits and 1 healthy control were used for histology.

The first phase of the study was designed to establish specific binding of targeted nanoparticles to integrins expressed in the diseased valves. It consisted of the following experimental groups: a "treatment" group (n = 7) which received 2.2 mL/kg of *α*_ν _*β*_3 _*integrin-targeted *perfluorocarbon nanoparticles, a control group (n = 6) which received 2.2 mL/kg of *untargeted *perfluorocarbon nanoparticles, and a "competition" group (n = 4) in which nonsignaling safflower nanoparticles (formulated with safflower oil in place of a fluorine core) were used as a pretreatment to establish targeting specificity by competitive inhibition of binding of a second dose of integrin targeted perfluorocarbon nanoparticles. These rabbits were pretreated with 2.2 mL/kg of α_ν_β_3 _integrin-targeted nanoparticles made with a nonsignaling safflower oil core, followed 2 hours later by 2.2 mL/kg of α_ν_β_3 _integrin-targeted perfluorocarbon nanoparticles. Three healthy control rabbits received 2.2 mL/kg of α_ν_β_3 _integrin-targeted perfluorocarbon nanoparticles. All contrast agents were injected intravenously into the ear vein through a 22G catheter.

Two hours after the final injection, the aortic valve leaflets were excised and preserved in formalin. ^19^F MR spectroscopy was performed on these valve leaflets at 11.7T.

In the second phase of the study, the aortic valves of four cholesterol-fed animals were used for ^19^F imaging at 11.7T. In each of these animals, α_ν_β_3 _integrin-targeted perfluorocarbon nanoparticles were administered as described above. To preserve the anatomic structure of these valves for imaging, the aortic roots were left partially intact. Immunohistochemistry was performed in two cholesterol-fed animals and one normal animal to verify the valvular pathology and display of targeted epitopes. All procedures were approved by the Washington University Animal Care Committee.

### Nanoparticle formulation

PFC nanoparticles were formulated using previously described methods [20]. Briefly, the nanoparticle emulsions comprised 20% (vol/vol) of either 15-crown-5 ether (Exfluor Research Corp., Round Rock, TX) or safflower seed oil (Sigma, #S8281), 2% (wt/vol) of a surfactant comixture, 1.8% (vol/vol) of glycerol (Sigma, #G9012), and distilled, deionized water. The surfactant comixture for the α_ν_β_3 _targeted nanoparticles contained 98.9 mole% of egg phosphatidylcholine (Avanti Polar Lipids Inc., # 840051P), 0.093 mole% peptidomimetic vitronectin antagonist (Kereos, Inc., St. Louis, MO), and 1.0 mole% of dipalmitoyl phosphotidylethanolamine (Avanti Polar Lipids Inc., # 850705). For the nontargeted nanoparticles, egg phosphatidylcholine and dipalmitoyl phosphotidylethanolamine were proportionally increased to replace the targeting ligand.

### MRI protocol

*Ex vivo *^19^F spectroscopy was performed to determine specificity of targeting and sensitivity to detection of a fluorine signal. An 11.7T Varian INOVA imaging console (Varian, Inc., Palo Alto, CA) with an 8 cm diameter horizontal bore was used. Spectra were acquired using a custom built 4-turn solenoid coil tuned to 470 MHz. A spin echo pulse sequence was used with TR = 2s, TE = 10ms, 512 signal averages, and a scan time of approximately 20 minutes. A spectrally distinct reference standard consisting of 2 μL of 1% nontargeted perfluoro-octyl-bromide (PFOB) nanoparticle emulsion was included with each sample to allow quantitative comparison of spectra.

After feasibility of spectroscopy and specificity of targeting were established,* ex vivo *^19^F imaging was performed on four specimens using a custom built 2-turn solenoid coil. For anatomical localization, proton images showing the cross-section of the basal aortic root and valve were acquired using a spin echo imaging sequence with TR = 1s, TE = 40ms, 0.5 mm slice thickness, 2 × 2 cm^2 ^field of view, 128 × 128 matrix (resulting in 0.16 × 0.16 mm^2 ^in-plane resolution), and 8 signal averages. After proton acquisition, the coil was tuned inside the magnet to 470 MHz and coregistered ^19^F projection images were acquired using a similar spin echo imaging sequence with TR = 0.5s, TE = 18ms, 2 × 2 cm^2 ^field of view, 16 × 16 matrix (resulting in 0.3 × 0.3 mm^2 ^in-plane resolution), and 128 signal averages. Scan time was approximately 30 minutes.

### Quantification of binding sites

The relationship between the PFOB and crown ether signals was defined by acquiring spectra from a series of phantoms containing varying volumes of PFOB and crown ether emulsions. Because a given volume of CE has a stronger signal than a given volume of PFOB, phantoms were prepared with a fixed 5:1 ratio between PFOB and CE concentrations. Larger volumes of diluted PFOB were prepared at concentrations of 20%, 10%, 5%, 2.5%, 1.25%, and 0.625%; corresponding volumes of diluted CE emulsion were prepared at concentrations of 4%, 2%, 1%, 0.5%, 0.25%, and 0.125%. Phantoms (44 μL) were prepared containing 2 μL each of diluted PFOB emulsion and diluted crown ether emulsion, and the balance consisted of deionized water. Three phantoms were prepared at each pair of concentrations and spectra were acquired using the same pulse sequence as above, with 128 signal averages and scan time of 5 minutes.

### Data analysis

Quantitative comparison of spectra was performed using in-house software created in Matlab (The MathWorks, Natick, MA) to numerically integrate the area under the crown ether peak and the area under the largest PFOB peak (located 8 ppm away), and compute the ratio of integrated crown ether signal to integrated PFOB signal. Area under the peak was used as the metric for quantification to reduce the influence of noise on the measurements. These ratios were then normalized to the average value for control cholesterol-fed animals treated with nontargeted nanoparticles. One-way Analysis of Variance (ANOVA) comparison was performed on the mean ^19^F signals among the four groups. Post-hoc groupwise testing was performed using the Tukey-Kramer HSD test. All statistical analyses were performed using JMP (Version 5.1, SAS Institute Inc., Cary, NC).

The ratio between CE and PFOB signals, computed as the pooled average ratio over the phantom data, was used to calculate the concentration of CE in the rabbit aortic valves from the known concentration in the reference standard. The percentage of specifically targeted nanoparticles was estimated based on the difference between the fluorine signal from the targeted nanoparticles and the nontargeted nanoparticles (assumed to represent the proportion of nonspecifically trapped nanoparticles). The total number of nanoparticles present was estimated based on a nominal particle volume computed from the measured nominal particle diameter.

### Histology and immunohistochemistry

The aortic root was dissected free of the heart with aortic valve leaflets intact, and frozen in Optimal Cutting Temperature (OCT) medium. All staining was performed on 5 μm transverse frozen sections of the aortic root and valves. Morphology was determined using routine hematoxylin and eosin staining. Immunohistochemistry for endothelial cells (CD31 primary antibody against PECAM, JC70A, DakoCytomation, Carpinteria, CA), neovasculature (LM609 primary antibody against α_ν_β_3 _integrin, Chemicon International, Temecula, CA), macrophages (RAM-11 mouse monoclonal anti-rabbit macrophage antibody, DakoCytomation, Carpinteria, CA), and myofibroblasts (1A4 primary antibody against α-smooth muscle actin, Dako, Carpinteria, CA) was performed using a VectaStain^® ^ABC kit (Novocastra, Newcastle, UK). Images were digitized under 100×–600× power with a Nikon E800 research microscope and a Nikon DXM1200 camera.

## Results

### Quantification of binding sites

The relationship between CE detected in the aortic valves and the PFOB reference standards was calibrated using ^19^F spectra acquired from three sets of phantoms containing known amounts of CE and PFOB nanoparticle emulsions. The ratios of CE to PFOB signal intensities were 5.7 ± 0.49, 5.6 ± 0.29, and 5.3 ± 0.25 for the phantoms containing, respectively, 2 μL each of 1% CE and 5% PFOB, 0.5% CE and 2.5% PFOB, and 0.25% CE and 1.25% PFOB. As expected, a one-way ANOVA showed no significant difference between the mean value for each set (F-value = 0.48; P = 0.64). The pooled mean of the ratio of signal between CE and PFOB was 5.5 ± 0.56.

### ^19^F spectroscopic detection of targeted nanoparticles at 11.7T

**Figure 1:**
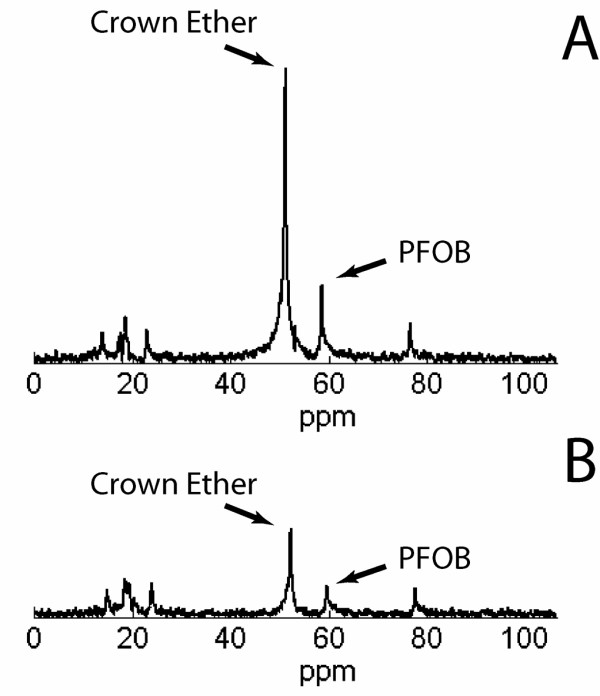
^**19**^**F spectra of valves – Representative CE spectra of valve leaflets from (A) a rabbit treated with targeted nanoparticles and (B) a rabbit treated with control untargeted nanoparticles.** The PFOB peaks correspond to the reference standard included for quantitative comparison. Notice the much stronger signal from the valve of the rabbit treated with targeted particles (CE/PFOB = 4.6) than from the valve of the rabbit treated with nontargeted particles (CE/PFOB = 2.2).

**Figure 2:**
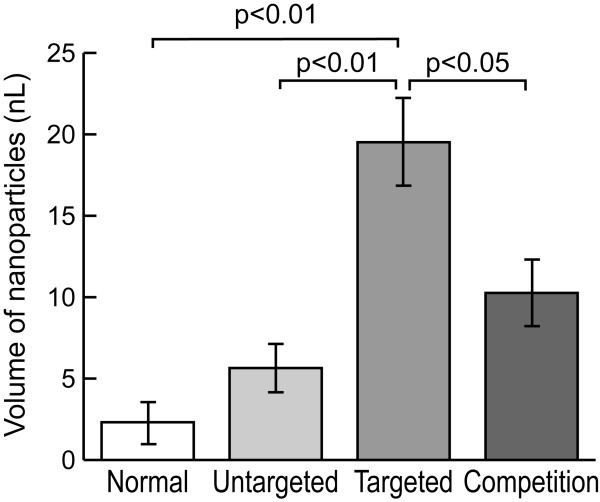
**Quantitative comparison of spectra – Comparison of CE spectra from the four study groups.** All values are nanoliters of emulsion present in the valve, as calculated from the ^19^F MR signal. Note that the valves treated with targeted particles have ~3× the signal of control valves and ~2× the signal of valves from the competitive inhibition group. Minimal nanoparticle deposition occurs in non-atherosclerotic animals treated with targeted nanoparticles, where no angiogenesis (and hence no binding ligands) are present in the valve.

The nanoparticle crown ether signal was readily detected from all samples undergoing fluorine spectroscopy. Representative spectra are shown in Figure 1. One-way ANOVA comparison of the mean ^19^F signals among the four groups indicated a significant difference, (F = 11.5, P < 0.001). The average volume of nanoparticles in valves in the treatment group receiving targeted nanoparticles was 19.5 ± 2.7 nL,3.5 times higher than the 5.6 ± 1.5 nL observed in the control group receiving untargeted nanoparticles (p < .01) (Figure 2). The average volume of nanoparticles in valves from the competitive inhibition group was 10.3 ± 2.0 nL, approximately half that of valves from the treatment group (p < 0.05). Valves from healthy rabbits treated with targeted nanoparticles contained an average of 2.3 ± 1.3 nL of nanoparticles, 8.5 times less than valves of rabbits with aortic valve disease treated with targeted nanoparticles (p < .001); this signal was not significantly different from the nanoparticle concentration in the control group.

In valves of animals treated with targeted nanoparticles, the estimated volume of nanoparticles present in the valve was 19.5 ± 2.7 nL, corresponding to 0.11% ± 0.01% of the total tissue volume. The average concentration of nanoparticles (and hence binding sites) was 1.8 × 10^11 ^± 0.24 × 10^11 ^particles per gram of tissue (a total of 7.0 × 10^8 ^± 0.96 × 10^8 ^particles in approximately 4 mg of tissue). As control nanoparticles did not contain a targeting ligand, the nanoparticles detected in those tissue samples were likely trapped or nonspecifically bound. A similar degree of nonspecific binding is expected for targeted nanoparticles, so it was estimated that approximately 70% of nanoparticles in the tissue samples containing targeted nanoparticles are specifically bound and approximately 30% are nonspecifically bound.

### ^19^F imaging of targeted nanoparticles

**Figure 3:**
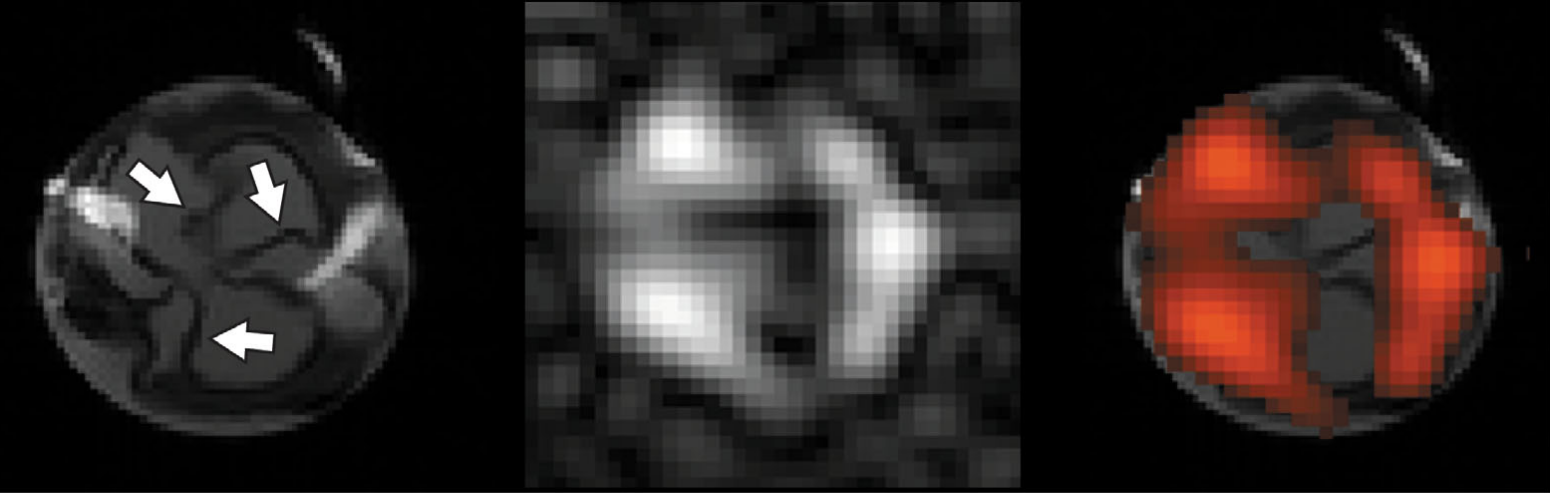
^**19**^**F imaging of the aortic valve – Coregistered proton (left) and fluorine (center) images of an aortic valve at 11.7T.** At far right, the fluorine image is false-colored and overlaid on the proton image, showing strong signal at the base of the valve leaflets, as well as signal from the body of the leaflets.

In four specimens, the perfluorocarbon component of targeted nanoparticles in the valve leaflets was successfully imaged. Co-registered proton and fluorine images were acquired, and the fluorine image was overlaid onto the proton image to form a "hot spot" image with anatomical localization of the fluorine signal (Figure 3). As expected, the greatest signal occurs at the base of the valve leaflets, but signal from the body of the leaflets is also visible.

### Histological analysis

**Figure 4:**
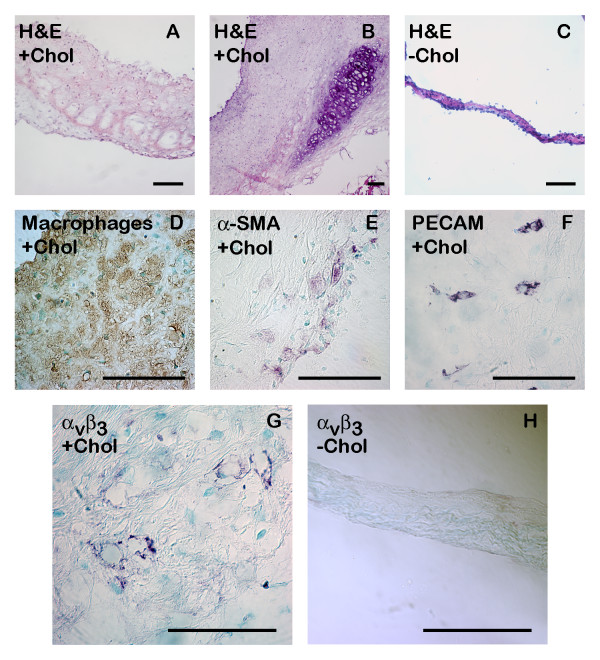
**Histological comparison of diseased and normal aortic valve leaflets – All scale bars are 50 μm in length. **(A, B): Hematoxylin & Eosin shows thickening and foam cell accumulation in diseased valve leaflets compared to normal valve leaflet (C). (B) shows bone formation at the base of a diseased aortic valve leaflet. The diseased valve leaflet contains extensive macrophage infiltration (D) and smooth muscle actin-positive myofibroblasts (E). Endothelial staining (PECAM) shows microvasculature within diseased valve leaflet (F). Macrophage, smooth muscle actin, and PECAM stains were negative in normal aortic valve (data not shown). α_ν_β_3 _integrin stain shows angiogenesis in diseased valve leaflet with highly upregulated expression of α_ν_β_3 _(G), while there is minimal expression in the healthy valve leaflet (H).

Histology of transverse sections of the aortic valve exhibited gross thickening and extensive foam cell infiltration of diseased valve leaflets compared to healthy leaflets, especially near the base (Figure 4A–C). Non-calcified bone was observed in the base of one valve leaflet (Figure 4B). Extensive macrophage infiltration was observed in the diseased valve (Figure 4D) as well as differentiation of cells into activated myofibroblasts on the aortic endothelial side of the valve (Figure 4E), suggesting an active inflammatory process. These cells were absent in the normal valve. Endothelial staining for PECAM confirmed the presence of abnormal microvessels within the diseased valve leaflets (Figure 4F), with no such vessels present in a normal valve. These vessels accumulated near the base of the valve leaflets, emanating from vascular beds in the aortic wall, similar to vasa vasorum penetrating into atherosclerotic plaque under cholesterol drive [21]. Staining for α_ν_β_3 _integrin confirmed that the observed abnormal microvessels have highly upregulated α_ν_β_3 _expression (Figure 4G), and can bind to the targeting ligand on the nanoparticles. Only minimal α_ν_β_3 _expression was observed in the normal valve (Figure 4H).

## Discussion

In this study we demonstrate specific binding of integrin-targeted nanoparticles to neovasculature in experimental valve disease, and provide proof of concept for their detection with magnetic resonance imaging and spectroscopy methods. Although the presence of angiogenesis in atherosclerotic plaques in the aorta of this model has been described previously [15], angiogenesis in the aortic valve has not been reported. We demonstrate that the model develops sufficient valvular inflammation and angiogenic expression of α_ν_β_3 _integrins to permit ^19^F spectroscopy and imaging *ex vivo*, and we have illustrated the presence of angiogenesis by immunohistochemistry. The sensitivity of this approach for molecular detection of sparse quantities of inflammatory epitopes in very thin structures at high field strengths establishes a basis for future efforts to develop localized spectroscopic methods at clinical field strengths that could be useful for detecting disease and monitoring therapies.

Inflammation and angiogenesis are a natural part of the body's wound healing response, but can become pathological in certain conditions, such as atherosclerosis [21,22] and cancer [23]. Inflammatory tissue components stimulate formation of neovasculature, which then serves as a conduit for deployment of cytokines and other inflammatory molecules, often creating a vicious cycle in disease evolution. Inflammation and angiogenesis are both manifest in early valve disease [6-9], which has led to reassessment of its early pathophysiology, (formerly regarded as a passive degenerative process). Indeed, early valve lesions bear striking similarities to early inflammatory atherosclerotic lesions, with infiltration by various cell types, including macrophages and T-cells, and upregulation of cytokines, integrins, and cellular adhesion molecules [5-9].

The α_ν_β_3 _integrin is a well described marker for angiogenesis [15], and it is upregulated on the lumenal side of neovascular endothelium, but only sparsely expressed on mature vascular endothelium. The targeting ligand used in this study is highly specific; it is a peptidomimetic small molecule which functions as a vitronectin antagonist with nanomolar K_d _values [24]. The fluorine signal from the valves of rabbits treated with α_ν_β_3_-targeted nanoparticles was more than triple that of the valves of rabbits treated with untargeted nanoparticles, consistent with the presence of specific neovasculature targets. Because the nanoparticles are too large (200 nm) to escape even "leaky" neovasculature [25], it is highly unlikely that other epitopes on vascular smooth muscle cells or interstitial inflammatory cells are being targeted. The R_1 _and R_2_, 1.31 s^-1 ^and 14.1 s^-1^, respectively, do not change upon nanoparticle binding [26], ensuring accurate signal comparison between rabbits treated with targeted and untargeted nanoparticles.

One aim of the study was to develop and implement *quantitative *techniques to assess the degree of inflammation in aortic valves. ^19^F MRS/MRI of aortic valve leaflets may offer advantages over traditional proton imaging, due to the unique signal generated by fluorine. Fluorine is relatively easy to detect by MR, and minimal fluorine occurs in biological tissues, so a fluorinated contrast agent gives a unique, directly measurable signal with virtually no background. This allows data to be acquired at a single time point and mitigates problems of low contrast-to-noise ratio inherent in agents used for proton MRI, which affect water relaxation to generate contrast indirectly, and hence require paired comparison of pre- and post-contrast datasets. Acquisition of a single unique signal becomes especially important when the goal is to detect very sparse epitopes, as in the present study, because the error introduced by multiple measurements may overwhelm a low percentage signal change.

Furthermore, because ^19^F spectroscopy of fluorinated nanoparticles is potentially quantitative [16], this technique may prove useful in estimating concentrations of bound nanoparticles. These concentrations are theoretically directly related to the number of molecular binding sites in the valves. We estimate that, in the rabbits studied, an average of at least 0.11% of the total valvular tissue volume is occupied by neovasculature: a small but appreciable amount that demonstrates the sensitivity of the measurements. This estimate represents the percentage of tissue occupied by nanoparticles, approximately 70% specifically bound and 30% nonspecifically trapped due to the endothelial permeability and retention (EPR) effect, and likely represents a lower bound on the proportion of valve tissue occupied by neovasculature. If shown to be applicable *in vivo *on larger samples of valve tissue, this approach could prove useful for quantitative longitudinal evaluation of disease progression.

We have additionally demonstrated the feasibility of ^19^F imaging at 11.7T. A projection image of the valve was acquired with 0.3 × 0.3 mm^2 ^in-plane resolution and approximately 20 minute scan time. The images show heterogeneous distribution of fluorine signal corresponding to neovasculature. The preponderance of signal occurs at the edges of the valve leaflets, consistent with histological observation of the location of neovasculature, which typically emanates from vascular beds in the aortic root.

We have recently reported several results illustrating fluorine detection at 1.5T for both molecular imaging and angiography [27,28], and clinical scanners are being developed at increasing field strengths (3T and 7T), which will provide even greater sensitivity.

### Limitations

The data for this study were acquired *ex vivo *as a first step to demonstrate that: 1) angiogenesis occurs within the valve leaflets under experimental conditions, 2) is detectable by MR fluorine spectroscopy, and 3) holds potential for imaging and quantification. The study was performed on formalin-fixed tissue, which might affect local ^1^H signals but is unlikely to affect ^19^F measurements since the perfluorocarbons are chemically inert and the lipid components of the particle unreactive. The techniques developed might be extended to the *in vivo *situation to enable noninvasive detection of angiogenesis in aortic valves. We have recently acquired *in vivo *^19^F images under other conditions with a resolution of 1 × 1 × 3 mm^3^ in 2–2.5 minutes [28]. Various approaches for localized spectroscopy can be applied to isolate the valve, along with cardiac and respiratory gating to compensate for bulk motion. Nanoparticle dosage and time between injection and imaging are two parameters which would require optimization in an *in vivo *situation; this was beyond the scope of the current study, designed to demonstrate proof of principle.

Immunohistochemistry was obtained from separate specimens than those which underwent MRI, so the presence of a direct correlation of angiogenesis and ^19^F signal could not be tested. However, the *in vivo *competition data demonstrates targeting specificity.

Furthermore, a correlation between the extent of angiogenesis and clinical diagnosis of disease severity has been established in human patients with mild to moderate valve disease [9].

## Conclusion

We suggest that these data point the way toward the use of molecular targeted contrast agents for elucidating certain fundamental pathological processes underlying valvular disease. Detection and monitoring strategies based on the underlying pathology may prove more effective than exclusive reliance on symptoms, signs, or traditional late stage imaging techniques for diagnosis and treatment. The question of what to image as a meaningful biomarker of early valve disease is yet unclear: a variety of molecules (integrins, adhesion molecules, signaling molecules, etc.) and cell types (myofibroblasts, T cells, etc.) are potentially targetable. Indeed, a variety of agents may be complementary in teasing out complex pathobiology. Macrophages have been correlated with disease activity [27], and are easily identified with constructs such as iron oxide nanoparticles. However, agents targeting neovasculature could also be quite useful since it has been shown that neovascular development is required for atherosclerotic lesion evolution [29]. We have demonstrated tools to quantify angiogenesis in aortic valve leaflets, which may permit more detailed study of its role in the progression of valvular lesions.

## Competing interests

S. Wickline and G. Lanza:

Kereos, Inc.: equity, consulting, board membership

Philips Medical Systems: Research contract

The authors declare that they have no competing interests.

## Authors' contributions

EAW carried out the MRI data acquisition, drafted the manuscript, and performed data and statistical analyses. JC designed the MRI methods and data-processing methods, and provided support for MRI data acquisition. JSA helped to design the animal model and supervised animal handling. HZ performed immunohistochemistry. GML assisted with study design, nanoparticle design, and coordination. SAW conceived of the study, reviewed all intermediate and final data, and participated in drafting the manuscript, All authors read and approved the final manuscript.
